# Supplementary material for: LSD1-mediated enhancer silencing attenuates retinoic acid signalling during pancreatic endocrine cell development
Source: Nat Commun. 2020 Apr 29;11:2082. doi: 10.1038/s41467-020-16017-x (PMC7190832; doi:10.1038/s41467-020-16017-x)
Supplement: Supplementary file 1 — Supplementary Information [file 41467_2020_16017_MOESM1_ESM.pdf]

LSD1-mediated enhancer silencing attenuates RA signalling during pancreatic endocrine cell development

Vinckier, et al.

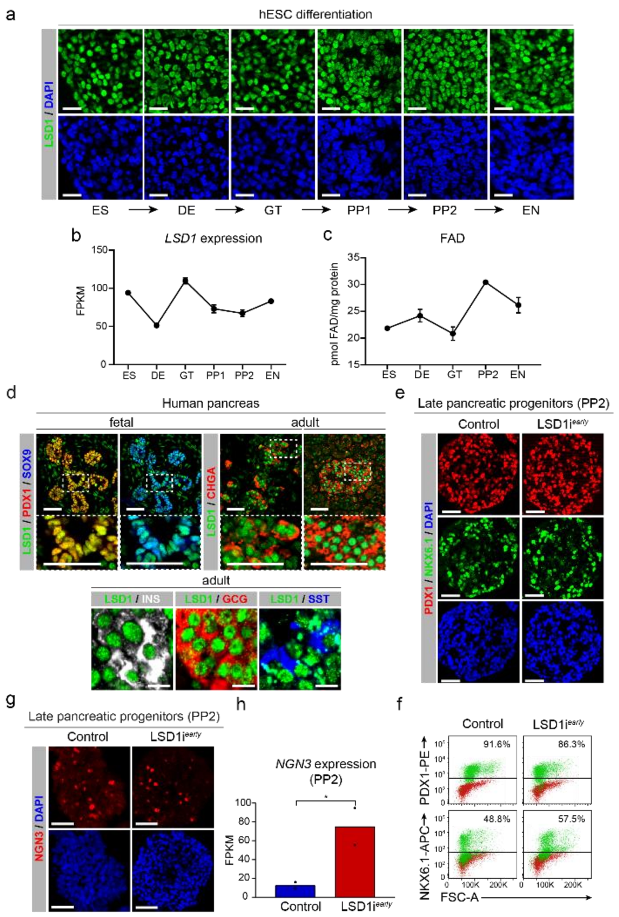

Supplementary Figure 1. Characterisation of LSD1 expression and effects of LSD1 inhibition on pancreatic progenitor cells. a, Immunofluorescent staining for LSD1 at each stage of hESC differentiation (representative image, n = 1 differentiation, three slides stained per stage). Scale bar, 25 µm. **b**, *LSD1* mRNA expression at each stage of differentiation determined by RNA-seq, measured in fragments per kilobase per million fragments mapped (FPKM). Data shown as mean FPKM ± S.E.M. (n = 3 replicates from independent differentiations; source data are provided as a Source Data file). **c**, FAD levels at the indicated stages of differentiation shown as mean ± S.E.M. (n = 3 technical replicates from one differentiation; source data are provided as a Source Data file). **d**, Immunofluorescent staining of pancreatic sections for LSD1 with the pancreatic progenitor markers PDX1 and SOX9 (55 days post-conception (dpc) embryonic pancreas; representative image, n = 1 embryo, three slides stained) or the pan-endocrine marker chromogranin A (CHGA) (94 dpc and adult pancreas; representative image, n = 1 embryo adult pancreas, respectively, three slides stained each). Scale bar, 50 µm (upper). Immunofluorescent staining for LSD1 with insulin (INS), glucagon (GCG) and somatostatin (SST) in adult human pancreas. Scale bar, 10 µm (lower). **e**, Immunofluorescent staining for NKX6.1 and PDX1 in control and LSD1i^{early} (TCP-treated) PP2 cells (representative images, n = 10 independent differentiations). Scale bar, 50 µm. **f**, Flow cytometry analysis for NKX6.1 and PDX1 comparing control and LSD1i^{early} PP2 cells. Isotype control for each antibody is shown in red and target protein staining in green. Percentage of cells expressing each protein is indicated (representative experiment, n = 2 independent differentiations). **g**, Immunofluorescent staining for NGN3 in control and LSD1i^{early} PP2 cells (representative images, n = 3 independent differentiations). Scale bar, 50 µm. **h**, *NGN3* mRNA levels in control and LSD1i^{early} PP2 cells. Data shown as mean FPKM ± S.E.M. (n = 2 replicates from independent differentiations; source data are provided as a Source Data file). *p.adj = 4.15 e-17, DESeq2. ES, human embryonic stem cells; DE, definitive endoderm; GT, primitive gut tube; PP1, early pancreatic progenitors; PP2, late pancreatic progenitors; EN, endocrine cell stage; FSC-A, forward scatter area.

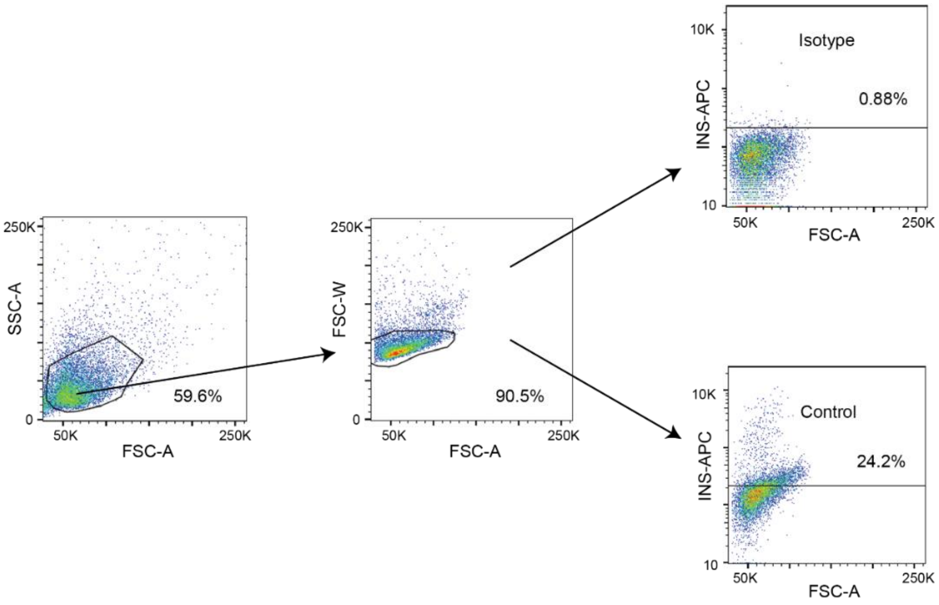

Supplementary Figure 2. Gating strategy for FACS analysis. Cells were gated to identify live cells (FSC-A vs SSC-A) and singlets (FSC-A vs FSC-W). Isotype controls were performed using antibodies against IgG conjugated to corresponding fluorophores.

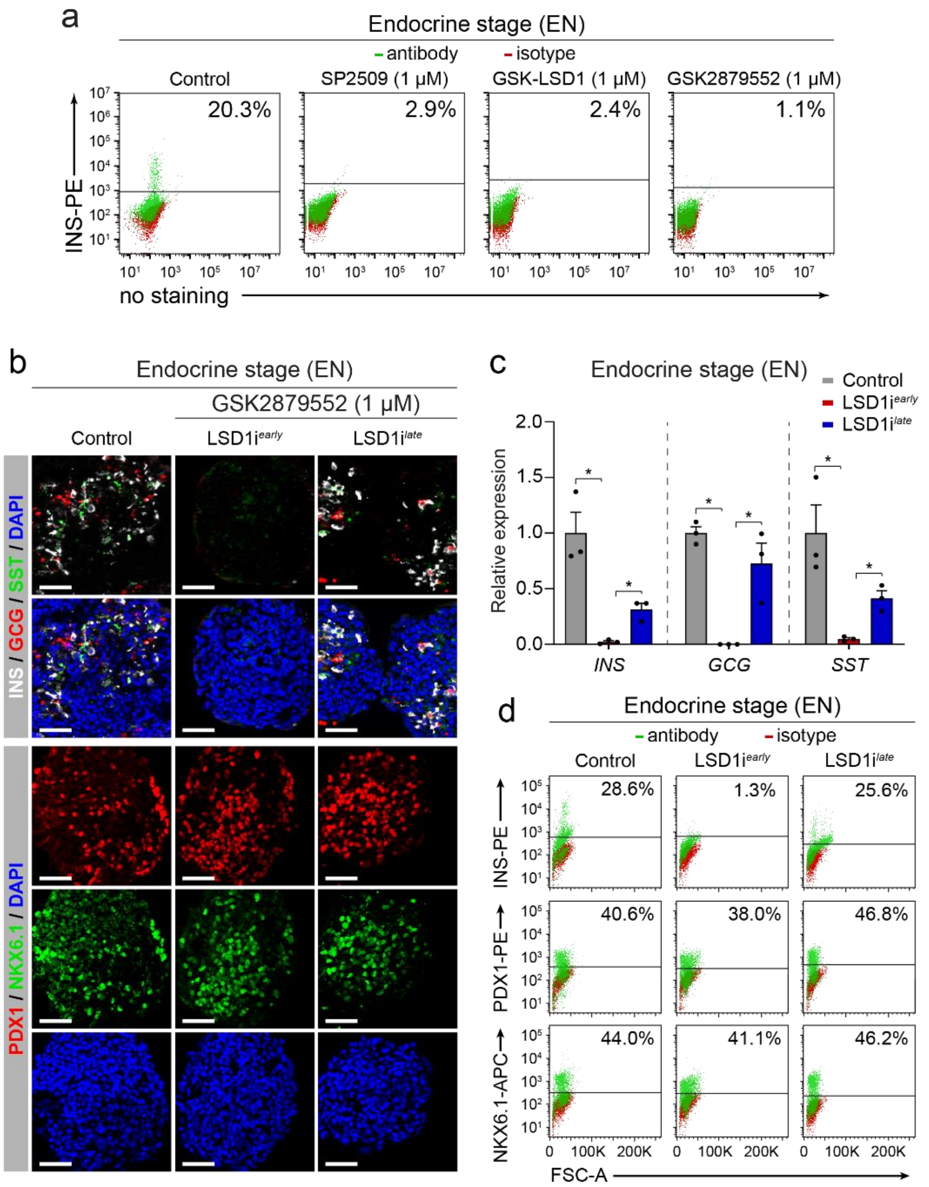

Supplementary Figure 3. Effects of different LSD1 inhibitors on endocrine cell differentiation. a, Flow cytometry analysis at EN stage for insulin (INS) comparing control cells to cells treated with the LSD1 inhibitors (SP2509 [1uM], GSK-LSD1 [1 μM] and GSK2879552 [1 μM]) during the PP1 to PP2 transition. Isotype control is shown in red and INS staining in green. Percentage of cells expressing INS is indicated (representative experiment, n = 2 independent differentiation). **b**, Immunofluorescent staining for pancreatic hormones INS, glucagon (GCG) and somatostatin (SST) or PDX1 and NKX6.1 in control EN cells compared to EN cells with early (LSD1i^{early}) and late (LSD1i^{late}) LSD1 inhibition mediated by GSK2879552 (representative images, n = 3 independent differentiations). Scale bar, 50 μm. **c**, qRT-PCR analysis for *INS*, *GCG* and *SST* in control, LSD1i^{early} and LSD1i^{late} EN cells, following GSK2879552-mediated LSD1 inhibition. Data are shown as mean ± S.E.M. (n = 3 replicates from independent differentiations with n = 3 technical replicates per sample; source data are provided as a Source Data file). P = 6.44 e-3, 6.36 e-3, 6.70 e-5, 1.74 e-2, 2.00 e-2, and 5.77 e-3, respectively, Student's t-test, 2 sided. **d**, Flow cytometry analysis at EN stage for NKX6.1, PDX1 and INS comparing control, LSD1i^{early} and LSD1i^{late} cells, following GSK2879552-mediated LSD1 inhibition. Isotype control for each antibody is shown in red and target protein staining in green. Percentage of cells expressing each protein is indicated (representative experiment, n = 2 independent differentiations). EN, endocrine cell stage; FSC-A, forward scatter area.

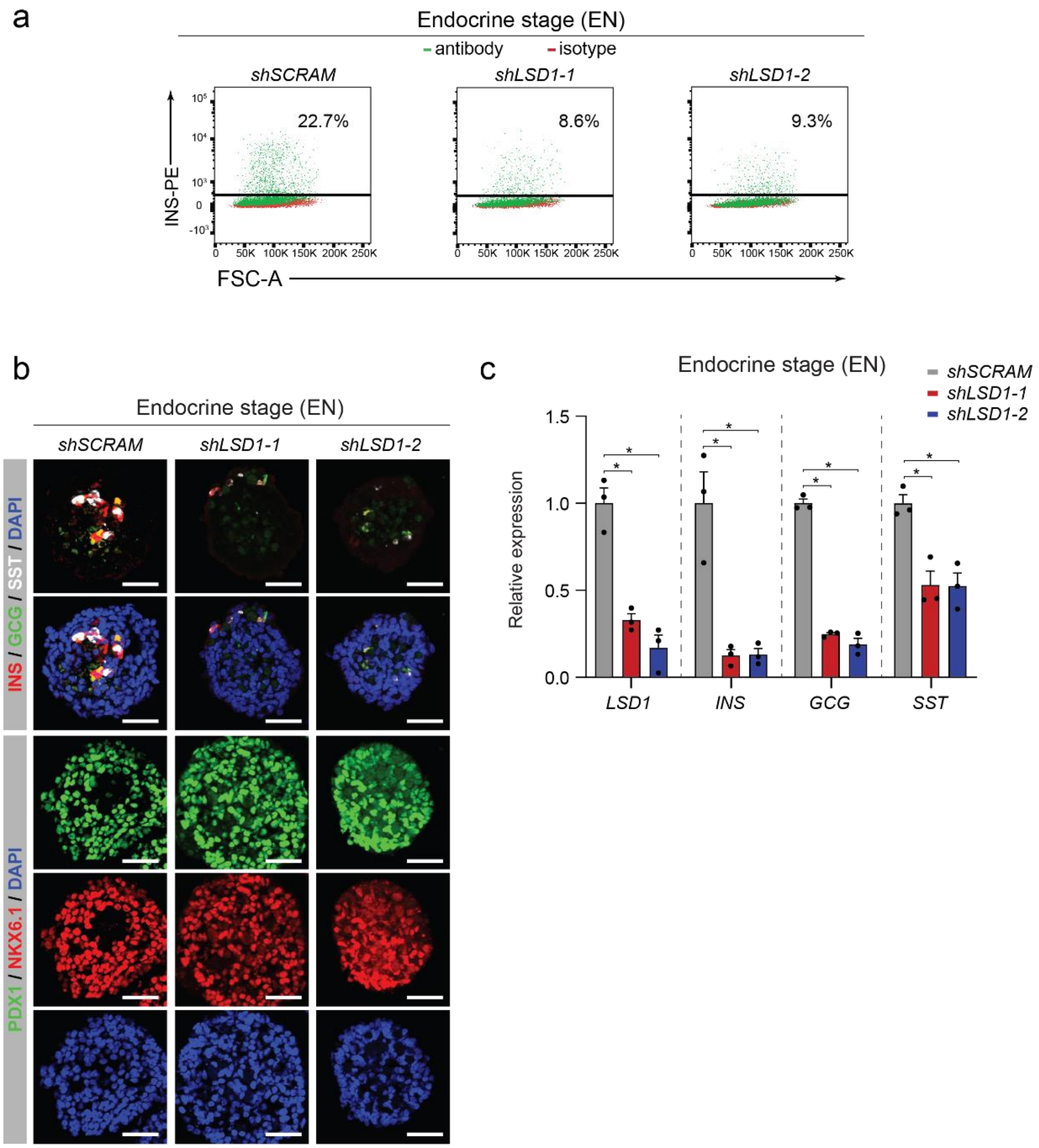

Supplementary Figure 4. Effects of shRNA-mediated *LSD1* knockdown on endocrine cell differentiation. a, Flow cytometry analysis at EN stage for insulin (INS) comparing scrambled control shRNA (*shSCRAM*) transduced cells to cells transduced with two different *LSD1* shRNA (*shLSD1-1* and *shLSD1-2*) lentiviral vectors at day 6 of differentiation (one day prior to PP1 stage). Isotype control is shown in red and INS staining in green. Percentage of cells expressing INS is indicated (representative experiment, n = 3 independent differentiations). **b**, Immunofluorescent staining for pancreatic hormones INS, glucagon (GCG) and somatostatin (SST) or PDX1 and NKX6.1 in *shSCRAM*-transduced EN cells compared to *shLSD1*-transduced EN cells. (representative image, n = 3 independent differentiations). Scale bar, 50 µm. **c**, qRT-PCR analysis for *LSD1*, *INS*, *GCG*, and *SST* in *shSCRAM*-transduced and s*hLSD1*-transduced EN cells. Data are shown as mean ± S.E.M. (n = 3 replicates from independent differentiations with n = 3 technical replicates per sample; source data are provided as a Source Data file). P = 2.16 e-3, 1.94 e-3, 8.99 e-3, 9.22 e-3, 8.00 e-6, 4.30 e-5, 7.66 e-3, and 6.23 e-3, respectively, Student's t-test, 2 sided. EN, endocrine cell stage; FSC-A, forward scatter area.

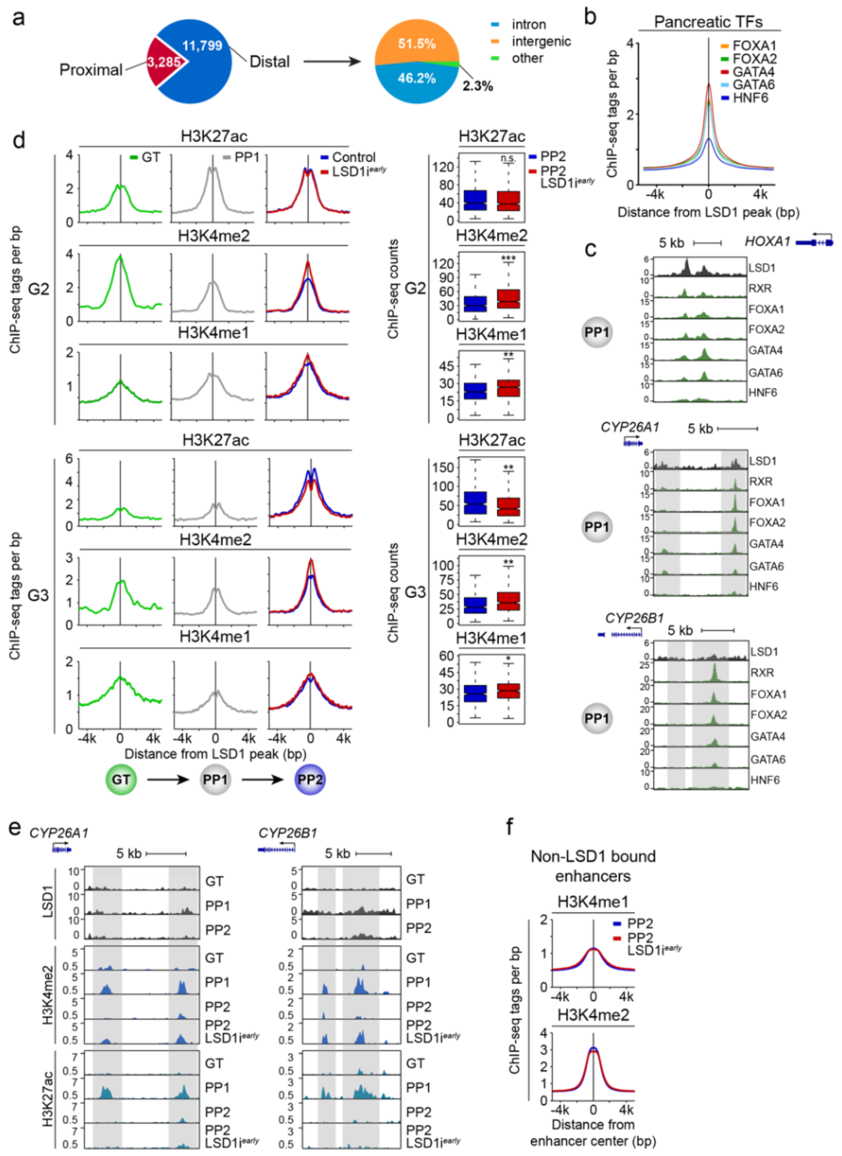

Supplementary Figure 5. Effects of LSD1 inhibition on chromatin state at distal genomics regions. **a**, LSD1 peak localisation across the genome relative to transcriptional start sites (TSSs). 15,084 total LSD1 peaks identified in PP1. 3,285 peaks are proximal (within 3kb of a TSS) and 11,799 distal (> 3kb from a TSS). **b**, Tag density plots displaying FOXA1, FOXA2, GATA4, GATA6, and HNF6 tag distribution at distal LSD1 peaks in PP1 cells. Plots are centred on LSD1 peaks. **c**, LSD1, FOXA1, FOXA2, GATA4, GATA6, HNF6, and RXR ChIP-seq profiles at enhancers near *HOXA1*, *CYP26A1* and *CYP26B1*. **d**, Tag density plots for G2 and G3 enhancers displaying H3K27ac, H3K4me2 and H3K4me1 tag distribution at PP1 and PP2 stage with and without early LSD1 inhibition (TCP, LSD1i^{early}, left). Plots are centred on PP1 LSD1 peaks. Box plots of H3K27ac, H3K4me2, and H3K4me1 ChIP-seq counts at G2 and G3 enhancers at PP2 stage with and without early LSD1 inhibition (LSD1i^{early}, right). Plots are centred on median, with box encompassing 25th-75th percentile and whiskers extending up to 1.5 interquartile range (Tukey style). P = 0.33, < 2.2 e-16, 8.5 e-6, 3.6 e-4, 3.9 e-11, and 5.0 e-4, respectively, Wilcoxon rank-sum test, 2 sided. **e**, LSD1, H3K4me2 and H3K27ac ChIP-seq profiles at enhancers near *CYP26A1* and *CYP26B1*. **f**, Tag density plots for PP1 enhancers (defined by H3K27ac peaks) not bound by LSD1 displaying H3K4me1 and H3K4me2 tag distribution at PP2 stage with and without early LSD1 inhibition (LSD1i^{early}). Plots are centred on PP1 enhancers not bound by LSD1. TF, transcription factor; GT, primitive gut tube; PP1, early pancreatic progenitors; PP2, late pancreatic progenitors. All ChIP-seq experiments, n = 2 replicates from independent differentiations, except for RXR which is n = 1.

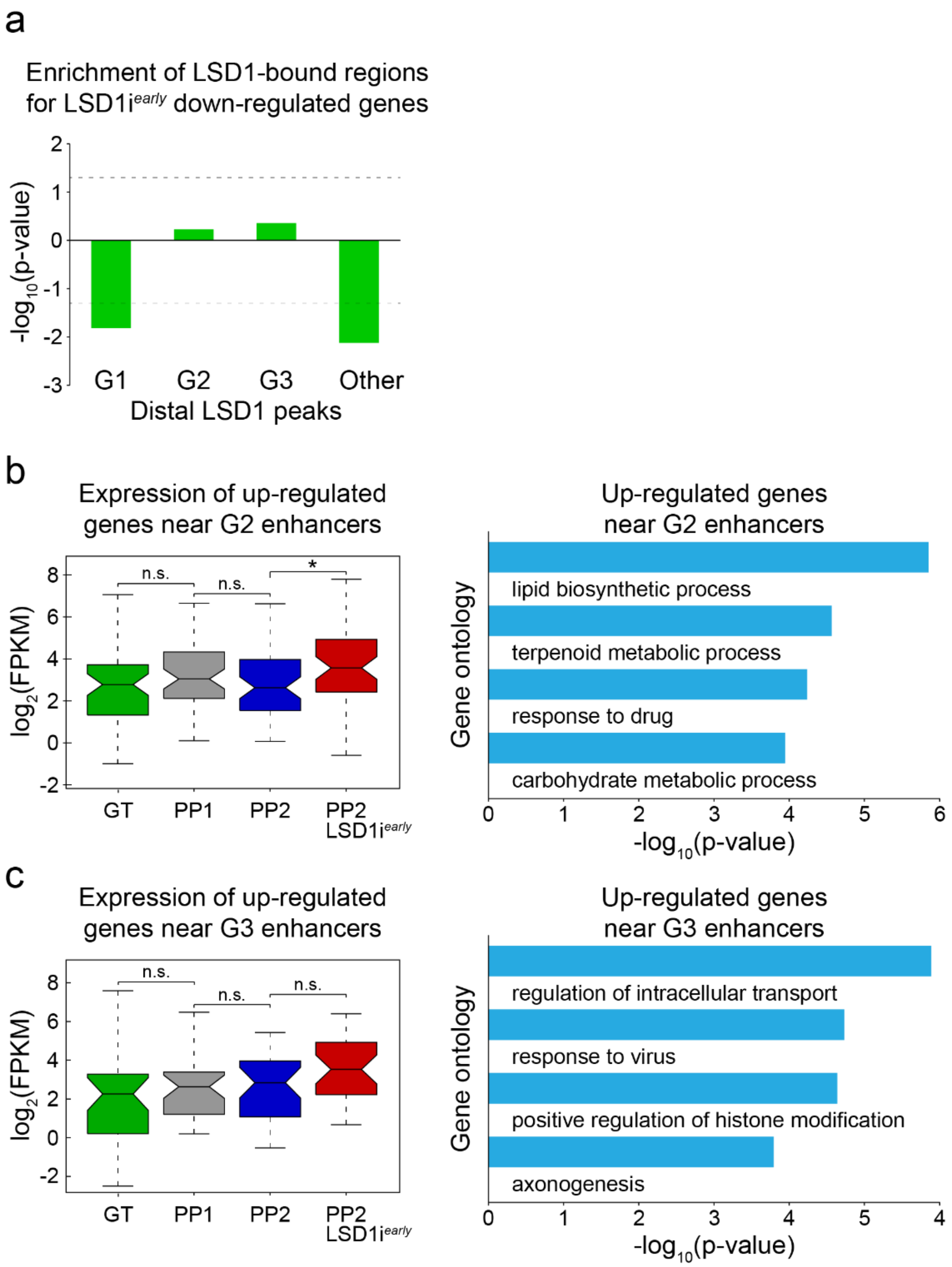

Supplementary Figure 6. Characterisation of genes dysregulated after LSD1 inhibition. a, Enrichment analysis of genes down-regulated by LSD1i^{early} (TCP) within 100kb of G1, G2 and G3 enhancers or other distal LSD1 peaks. Dashed lines indicate p-value = 0.05 for enrichment (positive value) or depletion (negative value). Fisher's exact test, 2 sided. **b**, Box plot of mRNA levels for 78 LSD1i^{early} up-regulated genes associated with G2 enhancers (left) and gene ontology analysis of those genes (right) (n = 2 replicates from independent differentiations). Plots are centred on median, with box encompassing 25th-75th percentile and whiskers extending up to 1.5 interquartile range (Tukey style). P = 0.68, 0.79, and 2.54 e-2, respectively, Wilcoxon rank-sum test, 2 sided. **c**, Box plot of mRNA levels for 53 LSD1i^{early} up-regulated genes associated with G3 enhancers (left) and gene ontology analysis of those genes (right) (n = 2 replicates from independent differentiations). Plots are centred on median, with box encompassing 25th-75th percentile and whiskers extending up to 1.5 interquartile range (Tukey style). P = 0.39, 0.33, and 0.09, respectively, Wilcoxon rank-sum test, 2 sided.

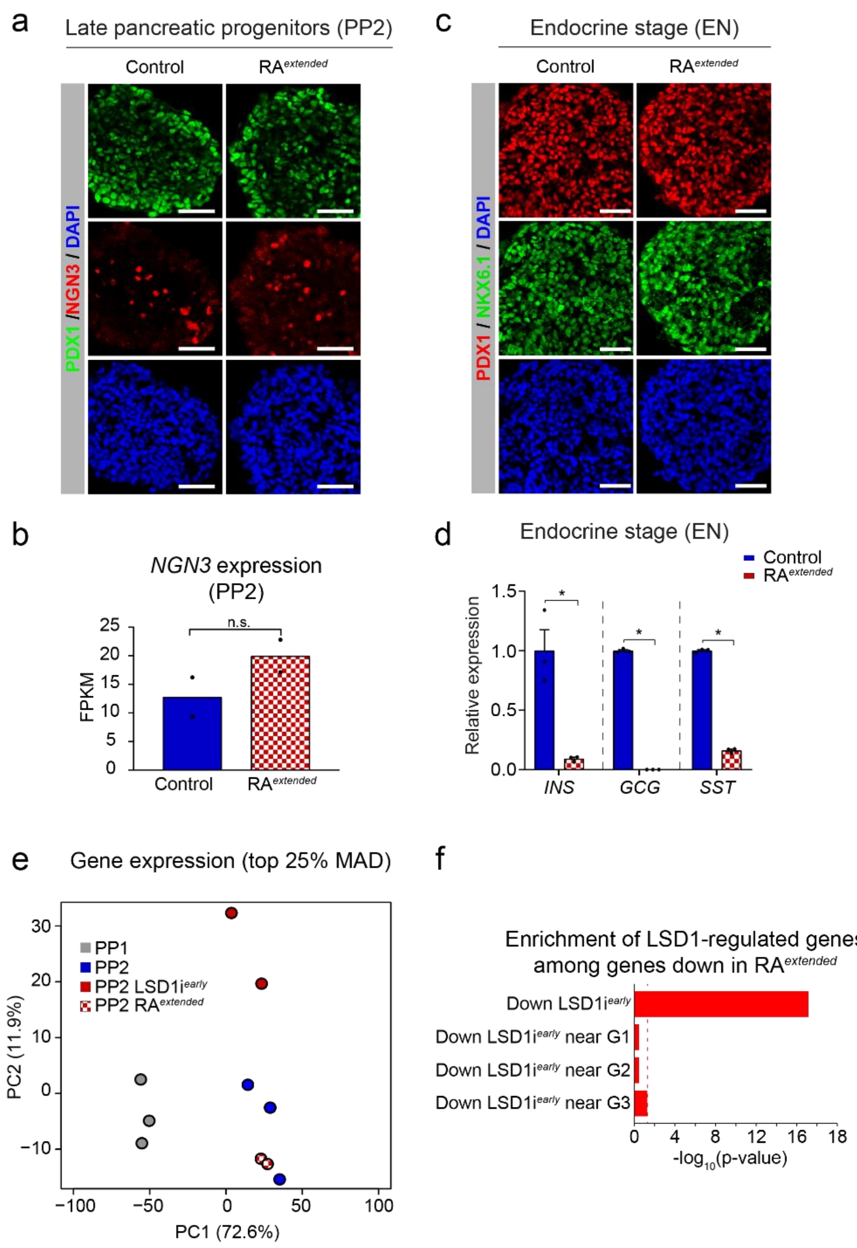

Supplementary Figure 7. Effects of prolonged retinoic acid treatment on pancreatic progenitor and endocrine cell phenotypes. a, Immunofluorescent staining for PDX1 and NGN3 in control and RA^{extended} PP2 cells (representative images, n = 3 independent differentiations). Scale bar, 50 µm. **b**, *NGN3* mRNA levels in control and RA^{extended} PP2 cells. Data shown as mean FPKM ± S.E.M. (n = 2 replicates from independent differentiations; source data are provided as a Source Data file). P = 0.39, DESeq2. **c**, Immunofluorescent staining for PDX1 and NKX6.1 in control EN compared to EN cells with RA^{extended} treatment (representative images, n = 3 independent differentiations). Scale bar, 50 µm. **d**, qRT-PCR analysis for *insulin* (*INS*), *glucagon* (*GLU*) and *somatostatin* (*SST*) in control and RA^{extended} endocrine stage (EN) cells. Data are shown as mean ± S.E.M. (n = 3 replicates from independent differentiations with n = 3 technical replicates per sample; source data are provided as a Source Data file). P = 6.88 e-3, < 1.0 e-6, and < 1.0 e-6, respectively, Student's t-test, 2 sided. **e**, Principal component (PC) analysis of the top 25% of variant genes determined by median absolute deviation (MAD) of expression values (FPKM) between samples. **f**, Enrichment analysis of genes associated with LSD1-bound enhancers and down-regulated by LSD1i^{early} (TCP) among those up-regulated by RA^{extended}. Dashed line indicates p-value = 0.05, Fisher's exact test.

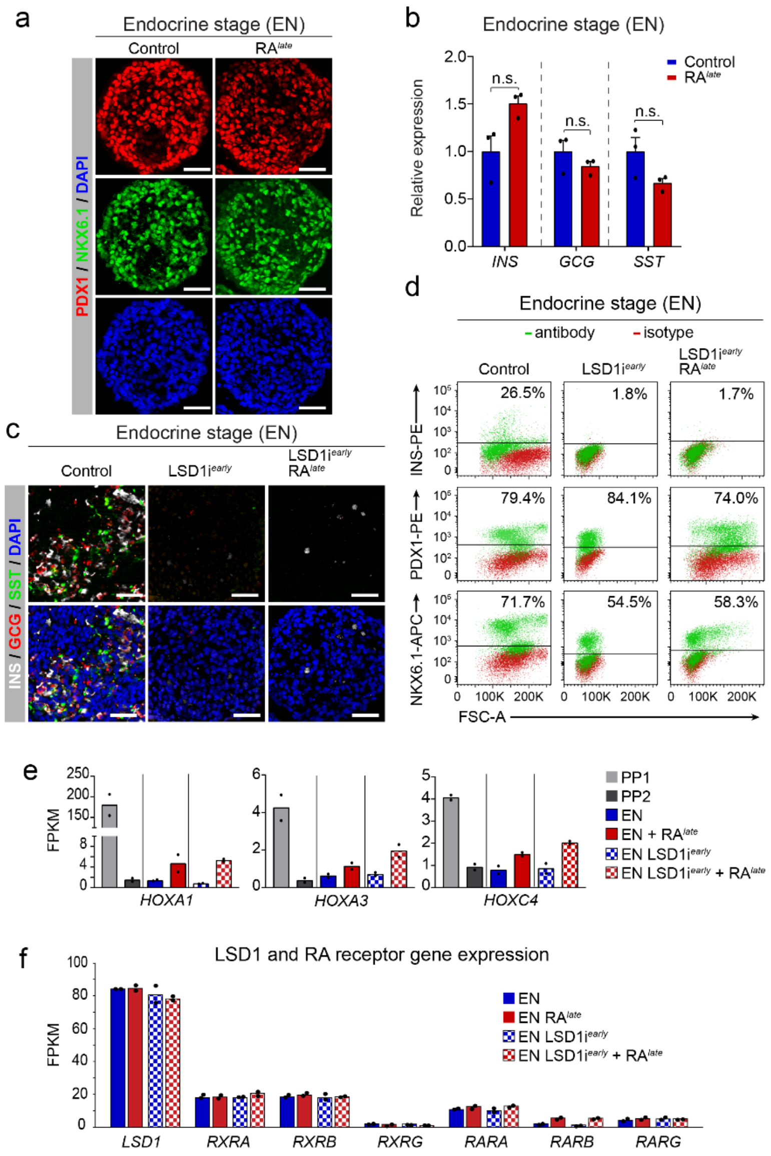

Supplementary Figure 8. Effects of re-introducing retinoic acid during endocrine cell differentiation with and without prior LSD1 inhibition. a, Immunofluorescent staining for PDX1 and NKX6.1 in control endocrine stage cells (EN) compared to EN cells with late retinoic acid (RA) treatment (RA*late*) (representative images, n = 3 independent differentiations). Scale bar, 50 µm. **b**, qRT-PCR analysis for *INS*, *GCG* and *SST* in control and RA*late* EN cells. Data are shown as mean ± S.E.M. (n = 3 replicates from independent differentiations with n = 3 technical replicates per sample; source data are provided as a Source Data file). P = 0.05, 0.29, and 0.10, respectively, Student's t-test, 2 sided. **c**, Immunofluorescent staining for INS, GCG and SST in control EN cells and EN cells with early LSD1 inhibition (TCP, LSD1i*early*) with and without late RA treatment (LSD1i*late* + RA*late*) (representative images, n = 3 independent differentiations). Scale bar, 50 µm. **d**, Flow cytometry analysis at EN stage for NKX6.1, PDX1 and INS comparing control EN cells to LSD1i*early* EN cells with and without late RA treatment (RA*late*). Isotype control for each antibody is shown in red and target protein staining in green. Percentage of cells expressing each protein is indicated (representative experiment, n = 2 independent differentiations). **e**, mRNA levels of *HOX* transcription factor genes in the indicated conditions. Data shown as mean FPKM ± S.E.M. (n = 2 replicates from independent differentiations; source data are provided as a Source Data file). **f**, mRNA levels of *LSD1* and RA receptor genes at EN stage. Data shown as mean FPKM ± S.E.M. (n = 2 replicates from independent differentiations; source data are provided as a Source Data file). INS, insulin; GCG, glucagon; SST, somatostatin; FSC-A, forward scatter area.

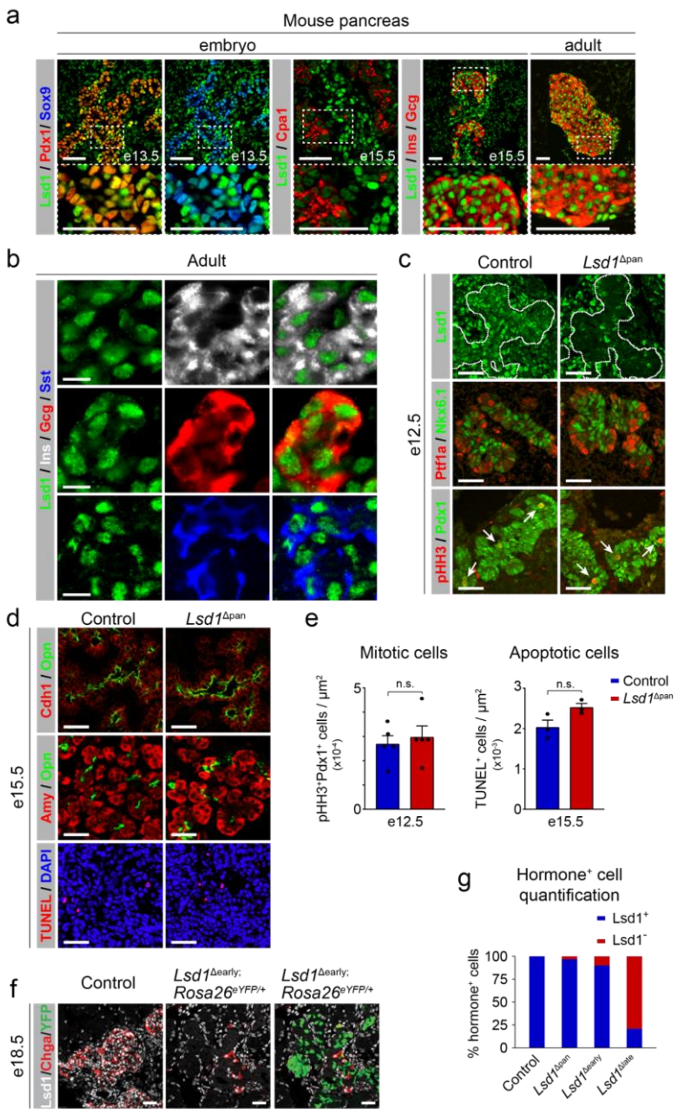

Supplementary Figure 9. Phenotypic characterisation of *Lsd1*$^{\Delta pan}$ mice. a, Immunofluorescent staining of embryonic (e) and adult mouse pancreas for Lsd1 with the pancreatic progenitor markers Pdx1 and Sox9, the acinar marker carboxypeptidase 1 (Cpa1) or insulin (Ins) and glucagon (Gcg). Boxed areas are shown in higher magnification (representative images, n = 3 embryos). Scale bar, 50 µm. **b**, Immunofluorescent staining of adult mouse pancreas for Lsd1 with Ins, Gcg and somatostatin (Sst) (representative images, n = 3 pancreata) Scale bar, 10 µm. **c**,**d** Immunofluorescent staining of pancreas from control and *Lsd1*$^{\Delta pan}$ embryos for Lsd1, Ptf1a, Nkx6.1, phospho-histone H3 (pHH3), and Pdx1 at e12.5 (c), or E-cadherin (Cdh1), osteopontin (Opn), amylase (Amy), and TUNEL at e15.5 (d). The nuclear counterstain, 4′,6-diamidino-2-phenylindole (DAPI) is shown together with TUNEL staining. Dashed lines delineate the pancreatic epithelium; arrows point to pHH3 and Pdx1 co-positive cells. Scale bar, 50 µm. **e**, Quantification of pHH3^{+} cells at e12.5 (n = 5 embryos per genotype; source data are provided as a Source Data file) and apoptotic cells (TUNEL^{+}) at e15.5 (n = 3 embryos per genotype) relative to pancreatic epithelial area. Data are shown means ± S.E.M. P = 0.63 and 0.07, respectively, Student's t-test, 2 sided. **f**, Immunofluorescent staining of pancreas from control and *Lsd1*$^{\Delta early}$;*Rosa26*$^{eYFP/+}$ mice for Lsd1, chromogranin A (Chga), and YFP at e18.5. Scale bar, 50 µm. **g**, Quantification of hormone^{+} cells staining positive for Lsd1 in control, *Lsd1*$^{\Delta pan}$, *Lsd1*$^{\Delta early}$, and *Lsd1*$^{\Delta late}$ mice at postnatal day (P) 0. A total of 864-1057 hormone^{+} cells (insulin^{+} or glucagon^{+}) were analysed per genotype and set as 100% (n = 3 embryos per genotype; source data are provided as a Source Data file).

Supplementary Tables

Supplementary Table 1. Pearson correlation coefficients of ChIP-seq replicates.

Condition	LSD1	H3K27ac	H3K4me1	H3K4me2	FOXA1	FOXA2	GATA4	GATA6	HNF6
GT	0.97	0.81	0.95	0.94	N/A	N/A	N/A	N/A	N/A
PP1	0.80	0.84	0.91	0.99	0.83	0.95	0.94	0.87	0.77
PP2	0.74	0.88	0.80	0.99	N/A	N/A	N/A	N/A	N/A
PP2 LSD1i^{early}	N/A	0.88	0.81	0.94	N/A	N/A	N/A	N/A	N/A

Supplementary Table 2. Primers used for RT-qPCR.

Primer Name	Sequence
INS-F	AAGAGGCCATCAAGCAGATCA
INS-R	CAGGAGGCGCATCCACA
GCG-F	AAGCATTTACTTTGTGGCTGGATT
GCG-R	TGATCTGGATTTCTCCTCTGTGTCT
SST-F	CCCCAGACTCCGTCAGTTTC
SST-R	TCCGTCTGGTTGGGTTCAG
TBP-F	ATTAAGGGAGGGAGTGGCAC
TBP-R	GCTTTGCTTCCCTTTCCCAA
LSD1-F	TGACCGGATGACTTCTCAAGA
LSD1-R	GTTGGAGAGTAGCCTCAAATGTC

Supplementary Table 3. Short hairpin sequences used for *LSD1* knockdown.

	Sense	Antisense
Scramble	5’-TGAACAAGATGAAGAGCACCTTCAAGAGAGGTGCTCTTCATCTTGTTCTTTTTTC-3’	5’-TCGAGAAAAAAGAACAAGATGAAGAGCACCTCTCTTGAAGGTGCTCTTCATCTTGTCA-3’
shLSD1-1	5’-CGGACAAGCTGTTCCTAAA-3’	5’-TTTAGGAACAGCTTGTCCG-3’
shLSD1-2	5’-GGAAGTTGTCATTCAGTTA-3’	5’-TAACTGAATGACAACTTCC-3’
