# Supplementary material for: LSD1-mediated enhancer silencing attenuates retinoic acid signalling during pancreatic endocrine cell development
Source: Nat Commun. 2020 Apr 29;11:2082. doi: 10.1038/s41467-020-16017-x (PMC7190832; doi:10.1038/s41467-020-16017-x)
Supplement: Supplementary file 24 — Reporting Summary [file 41467_2020_16017_MOESM24_ESM.pdf]

nature research

Corresponding author(s): Maike Sander

Reporting Summary

Nature Research wishes to improve the reproducibility of the work that we publish. This form provides structure for consistency and transparency in reporting. For further information on Nature Research policies, see Authors & Referees and the Editorial Policy Checklist.

Statistical parameters

When statistical analyses are reported, confirm that the following items are present in the relevant location (e.g. figure legend, table legend, main text, or Methods section).

n/a	Confirmed	
☐	☒	The exact sample size (*n*) for each experimental group/condition, given as a discrete number and unit of measurement
☐	☒	An indication of whether measurements were taken from distinct samples or whether the same sample was measured repeatedly
☐	☒	The statistical test(s) used AND whether they are one- or two-sided<br>*Only common tests should be described solely by name; describe more complex techniques in the Methods section.*
☐	☒	A description of all covariates tested
☐	☒	A description of any assumptions or corrections, such as tests of normality and adjustment for multiple comparisons
☐	☒	A full description of the statistics including central tendency (e.g. means) or other basic estimates (e.g. regression coefficient) AND variation (e.g. standard deviation) or associated estimates of uncertainty (e.g. confidence intervals)
☐	☒	For null hypothesis testing, the test statistic (e.g. *F*, *t*, *r*) with confidence intervals, effect sizes, degrees of freedom and *P* value noted<br>*Give P values as exact values whenever suitable.*
☐	☒	For Bayesian analysis, information on the choice of priors and Markov chain Monte Carlo settings
☐	☒	For hierarchical and complex designs, identification of the appropriate level for tests and full reporting of outcomes
☐	☒	Estimates of effect sizes (e.g. Cohen's *d*, Pearson's *r*), indicating how they were calculated
☐	☒	Clearly defined error bars<br>*State explicitly what error bars represent (e.g. SD, SE, CI)*

Our web collection on statistics for biologists may be useful.

Software and code

Policy information about availability of computer code

Data collection	No software was used for data collection.

Data analysis	Bowtie2 v 2.2.7 was used for mapping of ChIP-seq data to the human reference genome hg19 with the option "--very-sensitive" and all other parameters set to defaults.<br>STAR v2.4.0f1 was used to map RNA-seq data to the human reference genome hg19 with default parameters.<br>Samtools v1.5 was used to remove reads marked as duplicates with default parameters.<br>The Bedtools suite v2.17.0 was used for ChIP-seq peak to gene proximity analysis. windowBed with the option "-w 50000" was used to find peaks within ± 50kb (100kb window) of gene transcription start sites. shuffleBed with the option "-chrom" was to randomize chromosomal regions, within the same chromosomes, for proximity enrichment analysis. The process was repeated 10,000 in order to determine p-values.<br>The HOMER suite of bioinformatics tools were used to call ChIP-seq peaks, annotate peaks, analyze RNA-seq data, perform differential peak and gene expression (invoking the R program DESeq2, version 1.26.0) analysis, and generate bigWig files for data visualization. All programs were run using default settings. Default FDR for HOMER ChIP-seq peak calling is ≤ 0.001.<br>FlowJo™ Software version 10 was used to analyze FACs data.<br>Figures were assembled using Adobe Creative Suite version 5.1.

For manuscripts utilizing custom algorithms or software that are central to the research but not yet described in published literature, software must be made available to editors/reviewers upon request. We strongly encourage code deposition in a community repository (e.g. GitHub). See the Nature Research guidelines for submitting code & software for further information.

Data

Policy information about availability of data

All manuscripts must include a data availability statement. This statement should provide the following information, where applicable:

- Accession codes, unique identifiers, or web links for publicly available datasets
- A list of figures that have associated raw data
- A description of any restrictions on data availability

The accession number for the ChIP-seq and RNA-seq data reported in this manuscript is GSE104840 (Figures 2,3,4,5 and Suppl. Figures 4,5,6,7).
The accession number for previously reported H3K4me1 and H3K27ac ChIP-seq data is GSE54471 (Figure 2 and Suppl. Figure 4).
The accession number for previously reported RNA-seq data is E-MTAB-1086 (Figure 3 and Suppl. Figures 1,5,6).

Field-specific reporting

Please select the best fit for your research. If you are not sure, read the appropriate sections before making your selection.

[x] Life sciences [ ] Behavioural & social sciences [ ] Ecological, evolutionary & environmental sciences

For a reference copy of the document with all sections, see nature.com/authors/policies/ReportingSummary-flat.pdf

Life sciences study design

All studies must disclose on these points even when the disclosure is negative.

Sample size	For all phenotypic analysis, consistent results across > 10 experimental replications was considered sufficient to ensure reproducibility. Two to three biological replicates for ChIP-seq and RNA-seq was determined to be sufficient based on the research community standards when the studies were performed. https://www.encodeproject.org/about/experiment-guidelines
Data exclusions	Only data derived from failed differentiations, as measured by IFC, qPCR and Flow cytometry for successful generation of endocrine cells in control (untreated) samples, was excluded as this data is not representative of a valid differentiation.
Replication	The experiment inhibiting LSD1 during pancreatic differentiation of hESCs was repeated > 10 times and always resulted in the same phenotype showing a complete lack of endocrine cells. Experiments exposing cells to RA were conducted three times with consistent results. In all treatments and perturbations of hESCs we observed the same phenotype across experiments. For NGS sequencing data from biological replicates the correlation coefficient between replicates was calculated: Pearson correlations = 0.81 – 0.99 for histone modification and = 0.74 - 0.95 for transcription factor ChIP-seq data; Spearman correlation >0.9 for RNA-seq data. For Pdx1Cre-mediated deletion of Lsd1 we analyzed >10 embryos form independent litters and observed a 100% penetrant phenotype.
Randomization	Since cells were treated with inhibitors or genes were genetically deleted, randomization was not possible.
Blinding	We did not have the personnel resources to blind this study.

Reporting for specific materials, systems and methods

Materials & experimental systems

n/a	Involved in the study
✗	Unique biological materials
	✗ Antibodies
	✗ Eukaryotic cell lines
✗	Palaeontology
	✗ Animals and other organisms
	✗ Human research participants

Methods

n/a	Involved in the study
	✗ ChIP-seq
	✗ Flow cytometry
✗	MRI-based neuroimaging

Antibodies

Antibodies used

Secondary (immunofluorescence (IFC) staining):
AlexaFluor 647-conjugated donkey anti-guinea pig Jackson ImmunoResearch Labs Cat# 706-605-148, RRID:AB_2340476
AlexaFluor 647-conjugated donkey anti-rabbit Jackson ImmunoResearch Labs Cat# 711-606-152, RRID:AB_2340625
Cy3-conjugated donkey anti-goat Jackson ImmunoResearch Labs Cat# 705-165-003, RRID:AB_2340411
Cy3-conjugated donkey anti-guinea pig Jackson ImmunoResearch Labs Cat# 706-165-148, RRID:AB_2340460
Cy3-conjugated donkey anti-sheep Jackson ImmunoResearch Labs Cat# 713-005-147, RRID:AB_2340704
Cy3-conjugated donkey anti-rat Jackson ImmunoResearch Labs Cat# 712-005-153, RRID:AB_2340631
DyLight 488-conjugated donkey anti-mouse Jackson ImmunoResearch Labs Cat# 715-545-151, RRID:AB_2341099
DyLight 488-conjugated donkey anti-rabbit Jackson ImmunoResearch Labs Cat# 711-485-152, RRID:AB_2492289

Primary (IFC staining):
rabbit anti-SOX9 Millipore Cat# AB5535, RRID:AB_2239761
rabbit anti-somatostatin (SST) Dako Cat# A0566 ,RRID:AB_10013726
rat anti-E-cadherin (Cdh1) Sigma Cat# U3254, RRID:AB_477600
rabbit anti-Ptf1a Beta Cell Biology Consortium, AB2153
rabbit anti-LSD1 Abcam Cat# ab17721, RRID:AB_443964
rabbit anti-Phospo-Histone3 (Ser10) (pHH3) Cell Signaling Cat# 9701, RRID:AB_331535
rabbit anti-pancreatic polypeptide Y (Ppy) Dako, A0619
goat anti-carboxypeptidase A1 (Cpa1) R&D systems Cat# AF2765, RRID:AB_2085841
goat anti-ghrelin (Ghrl) Santa Cruz Cat# sc-10368, RRID:AB_2232479
goat anti-glucagon (Gcg) Santa Cruz Cat# sc-7780, RRID:AB_641025
goat anti-osteopontin (Opn) R&D systems Cat# AF808, RRID:AB_2194992
goat anti-PDX1 Abcam Cat# ab47383, RRID:AB_2162359
guinea pig anti-insulin (INS) Dako Cat# A0564, RRID:AB_10013624
mouse anti-glucagon (GCG) Sigma Cat# G2654, RRID:AB_259852
mouse anti-NKX6.1 Developmental Studies Hybridoma Bank Cat# F64A6B4, RRID:AB_532380
rabbit anti-amylase (Amy) Sigma Cat# A8273, RRID:AB_258380
rabbit anti-chromogranin A (CHGA) Dako Cat# IR502, RRID:AB_2341229
sheep anti-Neurogenin-3 R&D Systems Cat# AF3444, RRID:AB_2149527
guinea pig anti-Neurogenin-3, PMID: 15944193 (Henseleit et al. 2005)
rat anti-EpCAM Developmental Studies Hybridoma Bank Cat# G8.8, RRID:AB_2098655

Flow Cytometry:
mouse anti-PDX1-PE BD Biosciences Cat# 562161, RRID:AB_10893589
rabbit anti-INS-PE Cell Signaling Technology Cat# 8508S, RRID:AB_11179076
mouse anti-NKX6.1-Alexa Fluor® 647 BD Biosciences, 563338

ChIP-seq:
rabbit anti-H3K27ac Active Motif Cat# 39133, RRID:AB_2561016
rabbit anti-H3K4me1 Abcam Cat# ab8895, RRID:AB_306847
rabbit anti-H3K4me2 Millipore Cat# 07-030, RRID:AB_11213050
rabbit anti-LSD1 Abcam Cat# ab17721, RRID:AB_443964
goat anti-FOXA1 Abcam Cat# ab5089, RRID:AB_304744
goat anti-FOXA2 Santa Cruz Biotechnology Cat# sc-6554, RRID:AB_2262810
goat anti-GATA4 Santa Cruz Biotechnology Cat# sc-1237, RRID:AB_2108747
mouse anti-GATA6 Santa Cruz Biotechnology Cat# sc-9055, RRID:AB_2108768
rabbit anti-HNF6 Santa Cruz Biotechnology Cat# sc-13050, RRID:AB_2251852
rabbit anti-RXRA Santa Cruz Biotechnology Cat# sc-553, RRID:AB_2184874

Validation

Immunofluorescence (IFC) staining:

rabbit anti-SOX9 Millipore Cat# AB5535, RRID:AB_2239761
This antibody was validated in-house for IFC using mouse and human tissue (PMID: 21829703; PMID: 23318056). The antibody is highly specific for pancreatic ductal cells and pancreatic progenitors. The manufacture's website shows validation that the antibody is specific for both mouse and human SOX9 protein. The antibody is highly cited for IFC according to citeab.com.

rabbit anti-somatostatin (SST) Dako Cat# A0566 ,RRID:AB_10013726
This antibody was validated in-house for IFC using mouse and human tissue (PMID: 21266405; PMID: 23318056). The antibody is highly specific for a population of endocrine cells in the pancreatic islet. The manufacture's website shows validation that the antibody is specific for the human SST protein. The antibody is highly cited for IFC in mouse and human tissue according to citeab.com.

rat anti-E-cadherin (Cdh1) Sigma Cat# U3254, RRID:AB_477600
This antibody was validated in-house for IFC using mouse tissue (PMID: 21829703). The antibody is highly specific for epithelial cells in multiple tissues, including the pancreatic ductal cells and pancreatic progenitors. The manufacture's website shows validation that the antibody is specific for both mouse and human E-cadherin protein. The antibody is highly cited for IFC according to citeab.com.

rabbit anti-Ptf1a The Beta Cell Biology Consortium, AB2153
The manufacture's website shows validation that the antibody is specific for both mouse and human Ptf1a protein. The antibody was published for IFC in mouse tissue (PMID: 25371369).

rabbit anti-LSD1 Abcam Cat# ab17721, RRID:AB_443964
The manufacture's website shows validation that the antibody is specific for both mouse and human LSD1 protein. The antibody is highly cited for IFC according to citeab.com.

rabbit anti-Phospo-Histone3 (Ser10) (pHH3) Cell Signaling Cat# 9701, RRID:AB_331535
This antibody was validated in-house for IFC using mouse tissue (PMID: 26748698). The antibody is highly specific for cells undergoing mitosis. The manufacture's website shows validation that the antibody is specific for both mouse and pHH3 protein. The antibody is highly cited for IFC according to citeab.com.

rabbit anti-pancreatic polypeptide Y (PPY) Dako, A0619
This antibody was validated in-house for IFC using mouse tissue (PMID: 21266405). The antibody is highly specific for a small population of endocrine cells in the pancreatic islet. The manufacture's website shows validation that the antibody is specific for both mouse and PPY protein.

goat anti-carboxypeptidase A1 (Cpa1) R&D systems Cat# AF2765, RRID:AB_2085841
The manufacture's website shows validation that the antibody is specific for both mouse and human CPA1 protein. The antibody is highly cited for IFC according to citeab.com.

goat anti-ghrelin (Ghrl) Santa Cruz Cat# sc-10368, RRID:AB_2232479
The manufacture's website shows validation that the antibody is specific for both mouse and human GHRL protein. The antibody is highly cited for IFC according to citeab.com.

goat anti-glucagon (GCG) Santa Cruz Cat# sc-7780, RRID:AB_641025
This antibody was validated in-house for IFC using human tissue (PMID: 23318056). The antibody is highly specific for a population of endocrine cells in the pancreatic islet. The manufacture's website shows validation that the antibody is specific for both mouse and human GCG protein. The antibody is highly cited for IFC according to the manufacture's website.

goat anti-osteopontin (OPN) R&D systems Cat# AF808, RRID:AB_2194992
This antibody was validated in-house for IFC using mouse tissue (PMID: 21266405). The antibody is highly specific for a pancreatic ductal cells. The manufacture's website shows validation that the antibody is specific for the human OPN protein. The antibody is highly cited for IFC according to citeab.com.

goat anti-PDX1 Abcam Cat# ab47383, RRID:AB_2162359
The manufacture's website shows validation that the antibody is specific for both mouse and human PDX1 protein. The antibody is highly cited for IFC according to citeab.com.

guinea pig anti-insulin (INS) Dako Cat# A0564, RRID:AB_10013624
This antibody was validated in-house for IFC using mouse and human tissue (PMID: 21829703; PMID: 23318056). The antibody is highly specific for endocrine cells in the pancreatic islet. The manufacture's website shows validation that the antibody is specific for both mouse and human INS protein. The antibody is highly cited for IFC according to citeab.com.

mouse anti-glucagon (GCG) Sigma Cat# G2654, RRID:AB_259852
This antibody was validated in-house for IFC using human tissue (PMID: 23318056). The antibody is highly specific for a population of endocrine cells in the pancreatic islet. The manufacture's website shows validation that the antibody is specific for both mouse and human GCG protein. The antibody is highly cited for IFC according to the manufacture's website.

mouse anti-NKX6.1 Developmental Studies Hybridoma Bank Cat# F64A6B4, RRID:AB_532380

The manufacture's website shows validation that the antibody is specific for both mouse and human NKX6.1 protein. The antibody is highly cited for IFC according to citeab.com.

rabbit anti-amylase (AMY) Sigma Cat# A8273, RRID:AB_258380
This antibody was validated in-house for IFC using mouse tissue (PMID: 21266405). The antibody is highly specific for acinar cells in the pancreas. The manufacture's website shows validation that the antibody is specific for both mouse and human AMY protein. The antibody is highly cited for IFC according to citeab.com.

rabbit anti-chromogranin A (CHGA) Dako Cat# IR502, RRID:AB_2341229
The manufacture's website shows validation that the antibody is specific for human CHGA protein and cites several studies in which the antibody was used for flow cytometry.

sheep anti-Neurogenin-3 R&D Systems Cat# AF3444, RRID:AB_2149527
The manufacture's website shows validation that the antibody is specific for human NGN3 protein. The antibody has been cited multiple times for IFC according to citeab.com.

guinea pig anti-Neurogenin-3, PMID: 15944193 (Henseleit et al. 2005)
The antibody was validated in-house for IFC using mouse tissue (PMID: 15944193). The antibody has been shared widely and has been cited widely.

rat anti-EpCAM Developmental Studies Hybridoma Bank Cat# G8.8, RRID:AB_2098655
The antibody shows epithelial cell-specific staining across many tissues. The antibody is highly cited for IFC according to citeab.com.

Flow Cytometry:
mouse anti-NKX6.1-Alexa Fluor® 647 BD Biosciences Cat# 563338, RRID:AB_2738144
The manufacture's website shows validation that the antibody is specific for both mouse and human NKX6.1 protein and cites several studies in which the antibody was used for flow cytometry.

mouse anti-PDX1-PE BD Biosciences Cat# 562161, RRID:AB_10893589
The manufacture's website shows validation that the antibody is specific for both mouse and human PDX1 protein and cites several studies in which the antibody was used for flow cytometry.

rabbit anti-INS-PE Cell Signaling Technology Cat# 8508S RRID:AB_11179076
The manufacture's website shows validation that the antibody is specific for mouse INS protein and cites several studies in which the antibody was used for flow cytometry. The antibody was validated in-house on human tissue against IFC for insulin.

ChIP:
rabbit anti-H3K27ac Active Motif Cat# 39133, RRID:AB_2561016
The manufacture's website shows validation that the antibody is specific for human H3K27ac protein. The antibody is highly cited for ChIP according to citeab.com and was validated by ENCODE.

rabbit anti-H3K4me1 Abcam Cat# ab8895, RRID:AB_306847
The manufacture's website shows validation that the antibody is specific for human H3K4me1 protein. The antibody is highly cited for ChIP according to citeab.com and was validated by ENCODE. Also recently rigorously evaluated for target specificity in PMID: 30244833.

rabbit anti-H3K4me2 Millipore Cat# 07-030, RRID:AB_11213050
The manufacture's website shows validation that the antibody is specific for human H3K4me2 protein. The antibody is highly cited for ChIP according to citeab.com and was validated by ENCODE. Also recently rigorously evaluated for target specificity in PMID: 30244833.

rabbit anti-LSD1 Abcam Cat# ab17721, RRID:AB_443964
The manufacture's website shows validation that the antibody is specific for both mouse and human LSD1 protein. The antibody is highly cited for ChIP according to citeab.com.

goat anti-FOXA1 Abcam Cat# ab5089, RRID:AB_304744
The manufacture's website shows validation that the antibody is specific for both mouse and human FOXA1 protein. The antibody is highly cited for ChIP according to citeab.com.

goat anti-FOXA2 Santa Cruz Biotechnology Cat# sc-6554, RRID:AB_2262810
The manufacture's website shows validation that the antibody is specific for both mouse and human FOXA2 protein. The antibody is highly cited for ChIP and is being validated by ENCODE.

goat anti-GATA4 Santa Cruz Biotechnology Cat# sc-1237, RRID:AB_2108747
The manufacture's website shows validation that the antibody is specific for both mouse and human GATA4 protein. The antibody is highly cited for ChIP.

mouse anti-GATA6 Santa Cruz Biotechnology Cat# sc-9055, RRID:AB_2108768
The manufacture's website shows validation that the antibody is specific for both mouse and human GATA6 protein. The antibody has been validated in PMID:30982595.

rabbit anti-HNF6 Santa Cruz Biotechnology Cat# sc-13050, RRID:AB_2251852
The manufacture's website shows validation that the antibody is specific for human HNF6 protein. The antibody has been validated for ChIP in PMID:19129217.

rabbit anti-RXRA Santa Cruz Biotechnology Cat# sc-553, RRID:AB_2184874
The manufacture's website shows validation that the antibody is specific for human RXRA protein. The antibody has been validated for ChIP in PMID:26080448.

Eukaryotic cell lines

Policy information about cell lines

Cell line source(s)	CyT49 hESCs (provided by Viacyte Inc.), PMID: 18288110<br>HEK293T (ATCC® CRL-3216™)
Authentication	CyT49 human embryonic stem cells (hESC) experiments were conducted on early-passage (P29-P31) cryopreserved cells. All hESCs stocks were tested for mycoplasma and confirmed negative at the time of freezing. hESCs were karyotyped quarterly to ensure genomic stability and additionally subjected to Short Tandem Repeat (STR) profiling to authenticate cell line identity. In each experiment, proper differentiation of hESCs and purity of cultures was validated by flow cytometry analysis for stage-specific markers at the definitive endoderm, pancreatic progenitor and endocrine stages.
Mycoplasma contamination	Cell lines were tested for mycoplasma on a quarterly basis. No positive results were found.
Commonly misidentified lines<br>(See ICLAC register)	No commonly misidentified lines were used.

Animals and other organisms

Policy information about studies involving animals; ARRIVE guidelines recommended for reporting animal research

Laboratory animals	Mouse: Lsd1flox/+ (PMID: 17392792)<br>Mouse: Lsd1flox/flox (PMID: 17392792)<br>Mouse: Pdx1-Cre (PMID: 11973276)<br>Mouse: Pdx1-CreERTM (PMID: 11973276)<br>Mouse: Rosa26-eYFP (PMID: 11299042)<br>For timed matings, female mice were 8 to 16 weeks and male mice 8 weeks to 8 months of age.<br>Analysis was conducted on embryos of both sexes after timed matings. Midday on the day of vaginal plug appearance was considered embryonic day (e) 0.5.<br>All animal experiments were approved by the Institutional Animal Care and Use Committees of the University of California, San Diego. The animals were housed in a AAALAC accredited facility.
Wild animals	No wild animals were used in this study.
Field-collected samples	No field collected samples were used in this study.

Human research participants

Policy information about studies involving human research participants

Population characteristics	For this study human tissue was used that was obtained from the Birth Defects Research Laboratory of the University of Washington (embryomic pancreatic tissue) or the Network for Pancreatic Organ Donors with Diabetes (adult pancreata). The tissue was deidentified and population characteristics were not provided.
Recruitment	We did not recruit human subjects for this study.

ChIP-seq

Data deposition

- [x] Confirm that both raw and final processed data have been deposited in a public database such as GEO.
- [x] Confirm that you have deposited or provided access to graph files (e.g. BED files) for the called peaks.

Data access links
May remain private before publication.

To review GEO accession GSE104840:
Go to https://www.ncbi.nlm.nih.gov/geo/query/acc.cgi?acc=GSE104840
Enter token ezsrqwmwjpurxwx into the box

Files in database submission

ChIP-seq_H3K27ac_PP1_r1.bigWig
ChIP-seq_H3K27ac_PP2_r1.bigWig
ChIP-seq_H3K27ac_PP2_r2.bigWig
ChIP-seq_H3K27ac_PP2-LSD1-inh_r1.bigWig
ChIP-seq_H3K27ac_PP2-LSD1-inh_r2.bigWig
ChIP-seq_H3K4me1_PP1_r1.bigWig
ChIP-seq_H3K4me1_PP1_r2.bigWig
ChIP-seq_H3K4me1_PP2_r1.bigWig
ChIP-seq_H3K4me1_PP2_r2.bigWig
ChIP-seq_H3K4me1_PP2-LSD1-inh_r1.bigWig
ChIP-seq_H3K4me1_PP2-LSD1-inh_r2.bigWig
ChIP-seq_H3K4me2_GT_r1.bigWig
ChIP-seq_H3K4me2_PP1_r1.bigWig
ChIP-seq_H3K4me2_PP1_r2.bigWig
ChIP-seq_H3K4me2_PP2_r1.bigWig
ChIP-seq_H3K4me2_PP2_r2.bigWig
ChIP-seq_H3K4me2_PP2-LSD1-inh_r1.bigWig
ChIP-seq_H3K4me2_PP2-LSD1-inh_r2.bigWig
ChIP-seq_LSD1_PP1_r1.bigWig
ChIP-seq_LSD1_PP1_r2.bigWig
ChIP-seq_LSD1_PP2_r1.bigWig
ChIP-seq_LSD1_PP2_r2.bigWig
ChIP-seq_LSD1_PP2-LSD1-inh_r1.bigWig
ChIP-seq_LSD1_PP2-LSD1-inh_r2.bigWig
ChIP-seq_input_PP1.bigWig
ChIP-seq_input_PP2_r1.bigWig
ChIP-seq_input_PP2_r2.bigWig
ChIP-seq_input_PP2-LSD1-inh.bigWig
ChIP-seq_input_pooled.bigWig
RNA-seq_EN-LSD1-inh-early_r1.bigWig
RNA-seq_EN-LSD1-inh-early_r2.bigWig
RNA-seq_EN-LSD1-inh-early_RA-late_r1.bigWig
RNA-seq_EN-LSD1-inh-early_RA-late_r2.bigWig
RNA-seq_EN_r1.bigWig
RNA-seq_EN_r2.bigWig
RNA-seq_EN_r3.bigWig
RNA-seq_EN_RA-late_r1.bigWig
RNA-seq_EN_RA-late_r2.bigWig
RNA-seq_EN_RA-late_r3.bigWig
RNA-seq_PP1_r1.bigWig
RNA-seq_PP1_r2.bigWig
RNA-seq_PP1_r3.bigWig
RNA-seq_PP2-LSD1-inh-early_r1.bigWig
RNA-seq_PP2-LSD1-inh-early_r2.bigWig
RNA_seq_PP2-LSDi-inh-early_r3.bigWig
RNA-seq_PP2_r1.bigWig
RNA-seq_PP2_r2.bigWig
RNA-seq_PP2_r3.bigWig
RNA-seq_PP2_RA-early_r1.bigWig
RNA-seq_PP2_RA-early_r2.bigWig
ChIP-seq_H3K27ac_PP1_r1.fastq.gz
ChIP-seq_H3K27ac_PP2_r1.fastq.gz
ChIP-seq_H3K27ac_PP2_r2.fastq.gz
ChIP-seq_H3K27ac_PP2-LSD1-inh_r1.fastq.gz
ChIP-seq_H3K27ac_PP2-LSD1-inh_r2.fastq.gz
ChIP-seq_H3K4me1_PP1_r1.fastq.gz
ChIP-seq_H3K4me1_PP1_r2.fastq.gz
ChIP-seq_H3K4me1_PP2_r1.fastq.gz
ChIP-seq_H3K4me1_PP2_r2.fastq.gz
ChIP-seq_H3K4me1_PP2-LSD1-inh_r1.fastq.gz
ChIP-seq_H3K4me1_PP2-LSD1-inh_r2.fastq.gz
ChIP-seq_H3K4me2_GT_r1.fastq.gz
ChIP-seq_H3K4me2_PP1_r1.fastq.gz

ChIP-seq_H3K4me2_PP1_r2.fastq.gz
ChIP-seq_H3K4me2_PP2_r1.fastq.gz
ChIP-seq_H3K4me2_PP2_r2.fastq.gz
ChIP-seq_H3K4me2_PP2-LSD1-inh_r1.fastq.gz
ChIP-seq_H3K4me2_PP2-LSD1-inh_r2.fastq.gz
ChIP-seq_LSD1_PP1_r1.fastq.gz
ChIP-seq_LSD1_PP1_r2.fastq.gz
ChIP-seq_LSD1_PP2_r1.fastq.gz
ChIP-seq_LSD1_PP2_r2.fastq.gz
ChIP-seq_LSD1_PP2-LSD1-inh_r1.fastq.gz
ChIP-seq_LSD1_PP2-LSD1-inh_r2.fastq.gz
ChIP-seq_input_PP1.fastq.gz
ChIP-seq_input_PP2_r1.fastq.gz
ChIP-seq_input_PP2_r2.fastq.gz
ChIP-seq_input_PP2-LSD1-inh.fastq.gz
ChIP-seq_input_pooled.fastq.gz
RNA-seq_EN-LSD1-inh-early_r1.fastq.gz
RNA-seq_EN-LSD1-inh-early_r2.fastq.gz
RNA-seq_EN-LSD1-inh-early_RA-late_r1.fastq.gz
RNA-seq_EN-LSD1-inh-early_RA-late_r2.fastq.gz
RNA-seq_EN_r1.fastq.gz
RNA-seq_EN_r2.fastq.gz
RNA-seq_EN_r3.fastq.gz
RNA-seq_EN_RA-late_r1.fastq.gz
RNA-seq_EN_RA-late_r2.fastq.gz
RNA-seq_EN_RA-late_r3.fastq.gz
RNA-seq_PP1_r1.fastq.gz
RNA-seq_PP1_r2.fastq.gz
RNA-seq_PP1_r3.fastq.gz
RNA-seq_PP2-LSD1-inh-early_r1.fastq.gz
RNA-seq_PP2-LSD1-inh-early_r2.fastq.gz
RNA_seq_PP2-LSDi-inh-early_r3.fastq.gz
RNA-seq_PP2_r1.fastq.gz
RNA-seq_PP2_r2.fastq.gz
RNA-seq_PP2_r3.fastq.gz
RNA-seq_PP2_RA-early_r1.fastq.gz
RNA-seq_PP2_RA-early_r2.fastq.gz

Genome browser session
(e.g. UCSC)

http://genome.ucsc.edu/cgi-bin/hgTracks?db=hg19&lastVirtModeType=default&lastVirtModeExtraState=&virtModeType=default&virtMode=0&nonVirtPosition=&position=chr7%3A27088940%2D27179299&hgsid=782163733_6ASejNfHNK3NnASaOGDAPniNMcHb

Methodology

Replicates

Two biological replicates were used for ChIP-seq data analysis. Initial biological replicates used for peak calling were correlated with later biological replicates using Pearson correlation (see Methods for correlation table).

Sequencing depth

sample: ChIP-seq_H3K27ac_PP1_r1.fastq.gz, total number of reads: 53222837, uniquely mapped reads: 46978574, read length: 50bp, type: single-end
sample: ChIP-seq_H3K27ac_PP2-LSD1-inh_r1.fastq.gz, total number of reads: 44677011, uniquely mapped reads: 39132369, read length: 50bp, type: single-end
sample: ChIP-seq_H3K27ac_PP2-LSD1-inh_r2.fastq.gz, total number of reads: 29852103, uniquely mapped reads: 21600276, read length: 50bp, type: single-end
sample: ChIP-seq_H3K27ac_PP2_r1.fastq.gz, total number of reads: 55861467, uniquely mapped reads: 49037216, read length: 50bp, type: single-end
sample: ChIP-seq_H3K27ac_PP2_r2.fastq.gz, total number of reads: 40742429, uniquely mapped reads: 28827341, read length: 50bp, type: single-end
sample: ChIP-seq_H3K4me1_PP1_r1.fastq.gz, total number of reads: 36743410, uniquely mapped reads: 32141422, read length: 50bp, type: single-end
sample: ChIP-seq_H3K4me1_PP1_r2.fastq.gz, total number of reads: 44152011, uniquely mapped reads: 34335204, read length: 50bp, type: single-end
sample: ChIP-seq_H3K4me1_PP2-LSD1-inh_r1.fastq.gz, total number of reads: 21482019, uniquely mapped reads: 19010501, read length: 50bp, type: single-end
sample: ChIP-seq_H3K4me1_PP2-LSD1-inh_r2.fastq.gz, total number of reads: 39509255, uniquely mapped reads: 31126741, read length: 50bp, type: single-end
sample: ChIP-seq_H3K4me1_PP2_r1.fastq.gz, total number of reads: 27623401, uniquely mapped reads: 24364513, read length: 50bp, type: single-end
sample: ChIP-seq_H3K4me1_PP2_r2.fastq.gz, total number of reads: 29698479, uniquely mapped reads: 23128567, read length: 50bp, type: single-end
sample: ChIP-seq_H3K4me2_GT_r1.fastq.gz, total number of reads: 30280208, uniquely mapped reads: 21201295, read

length: 50bp, type: single-end
sample: RNA-seq_EN-LSD1-inh-early_r1.fastq.gz, total number of reads: 33715501, read length: 50bp, type: single-end
sample: RNA-seq_EN-LSD1-inh-early_r2.fastq.gz, total number of reads: 50136743, read length: 50bp, type: single-end
sample: RNA-seq_EN-LSD1-inh-early_RA-late_r1.fastq.gz, total number of reads: 37612651, read length: 50bp, type: single-end
sample: RNA-seq_EN-LSD1-inh-early_RA-late_r1.fastq.gz, total number of reads: 47717711, read length: 50bp, type: single-end
sample: RNA-seq_EN_r1.fastq.gz, total number of reads: 57855157, read length: 50bp, type: single-end
sample: RNA-seq_EN_r2.fastq.gz, total number of reads: 50500834, read length: 50bp, type: single-end
sample: RNA-seq_EN_r3.fastq.gz, total number of reads: 49859134, read length: 50bp, type: single-end
sample: RNA-seq_EN_RA-late_r1.fastq.gz, total number of reads: 49256438, read length: 50bp, type: single-end
sample: RNA-seq_EN_RA-late_r2.fastq.gz, total number of reads: 54375482, read length: 50bp, type: single-end
sample: RNA-seq_EN_RA-late_r3.fastq.gz, total number of reads: 56492842, read length: 50bp, type: single-end
sample: RNA-seq_PP1_r1.fastq.gz, total number of reads: 57537858, read length: 50bp, type: single-end
sample: RNA-seq_PP1_r2.fastq.gz, total number of reads: 39628784, read length: 50bp, type: single-end
sample: RNA-seq_PP1_r3.fastq.gz, total number of reads: 41736836, read length: 50bp, type: single-end
sample: RNA-seq_PP2-LSD1-inh-early_r1.fastq.gz, total number of reads: 171790467, read length: 50bp, type: single-end
sample: RNA-seq_PP2-LSD1-inh-early_r2.fastq.gz, total number of reads: 48681530, read length: 50bp, type: single-end
sample: RNA-seq_PP2-LSD1-inh-early_r3.fastq.gz, total number of reads: 50083394, read length: 50bp, type: single-end
sample: RNA-seq_PP2_r1.fastq.gz, total number of reads: 61081379, read length: 50bp, type: single-end
sample: RNA-seq_PP2_r2.fastq.gz, total number of reads: 31747065, read length: 50bp, type: single-end
sample: RNA-seq_PP2_r3.fastq.gz, total number of reads: 201378250, read length: 50bp, type: single-end
sample: RNA-seq_PP2_RA-early_r1.fastq.gz, total number of reads: 40051644, read length: 50bp, type: single-end
sample: RNA-seq_PP2_RA-early_r2.fastq.gz, total number of reads: 175734868, read length: 50bp, type: single-end
sample: ChIP-seq_H3K4me2_PP1_r1.fastq.gz, total number of reads: 22300436, uniquely mapped reads: 10843192, read length: 50bp, type: single-end
sample: ChIP-seq_H3K4me2_PP1_r2.fastq.gz, total number of reads: 56342893, uniquely mapped reads: 50159865, read length: 50bp, type: single-end
sample: ChIP-seq_H3K4me2_PP2-LSD1-inh_r1.fastq.gz, total number of reads: 46462096, uniquely mapped reads: 40782906, read length: 50bp, type: single-end
sample: ChIP-seq_H3K4me2_PP2-LSD1-inh_r2.fastq.gz, total number of reads: 33891993, uniquely mapped reads: 25958992, read length: 50bp, type: single-end
sample: ChIP-seq_H3K4me2_PP2_r1.fastq.gz, total number of reads: 21473335, uniquely mapped reads: 5102096, read length: 50bp, type: single-end
sample: ChIP-seq_H3K4me2_PP2_r2.fastq.gz, total number of reads: 55205606, uniquely mapped reads: 49456586, read length: 50bp, type: single-end
sample: ChIP-seq_input_pooled.fastq.gz, total number of reads: 34465886, uniquely mapped reads: 26038537, read length: 50bp, type: single-end
sample: ChIP-seq_input_PP1.fastq.gz, total number of reads: 60224090, uniquely mapped reads: 50370287, read length: 50bp, type: single-end
sample: ChIP-seq_input_PP2-LSD1-inh.fastq.gz, total number of reads: 40539036, uniquely mapped reads: 32855038, read length: 50bp, type: single-end
sample: ChIP-seq_input_PP2_r1.fastq.gz, total number of reads: 56948560, uniquely mapped reads: 47328667, read length: 50bp, type: single-end
sample: ChIP-seq_input_PP2_r2.fastq.gz, total number of reads: 56336917, uniquely mapped reads: 47528126, read length: 50bp, type: single-end
sample: ChIP-seq_LSD1_PP1_r1.fastq.gz, total number of reads: 53899255, uniquely mapped reads: 44504945, read length: 50bp, type: single-end
sample: ChIP-seq_LSD1_PP1_r2.fastq.gz, total number of reads: 32424236, uniquely mapped reads: 21876769, read length: 50bp, type: single-end
sample: ChIP-seq_LSD1_PP2-LSD1-inh_r1.fastq.gz, total number of reads: 38987477, uniquely mapped reads: 32001920, read length: 50bp, type: single-end
sample: ChIP-seq_LSD1_PP2-LSD1-inh_r2.fastq.gz, total number of reads: 47441068, uniquely mapped reads: 34650064, read length: 50bp, type: single-end
sample: ChIP-seq_LSD1_PP2_r1.fastq.gz, total number of reads: 53183208, uniquely mapped reads: 43080103, read length: 50bp, type: single-end
sample: ChIP-seq_LSD1_PP2_r2.fastq.gz, total number of reads: 64524268, uniquely mapped reads: 54390107, read length: 50bp, type: single-end
sample: ChIP-Seq_FOXA1_PP1_r1.fastq.gz, total number of reads: 50209121 uniquely mapped reads: 41845776 read length: 50bp, type: single-end
sample: ChIP-Seq_FOXA1_PP1_r2.fastq.gz, total number of reads: 47943675 uniquely mapped reads: 32925623 read length: 75bp, type: single-end
sample: ChIP-Seq_FOXA2_PP1_r1.fastq.gz, total number of reads: 36995538 uniquely mapped reads: 29376925 read length: 50bp, type: single-end
sample: ChIP-Seq_FOXA2_PP1_r2.fastq.gz, total number of reads: 38099987 uniquely mapped reads: 32071274 read length: 75bp, type: single-end
sample: ChIP-Seq_GATA4_PP1_r1.fastq.gz, total number of reads: 49059391 uniquely mapped reads: 43734397 read length: 75bp, type: single-end
sample: ChIP-Seq_GATA4_PP1_r2.fastq.gz, total number of reads: 28646736 uniquely mapped reads: 25250154read length: 75bp, type: single-end

sample: ChIP-Seq_GATA6_PP1_r1.fastq.gz, total number of reads: 47221769 uniquely mapped reads: 41628476 read length: 75bp, type: single-end
sample: ChIP-Seq_GATA6_PP1_r2.fastq.gz, total number of reads: 31474888 uniquely mapped reads: 27686309 read length: 75bp, type: single-end
sample: ChIP-Seq_HNF6_PP1_r1.fastq.gz, total number of reads: 47731079 uniquely mapped reads: 41721836 read length: 75bp, type: single-end
sample: ChIP-Seq_HNF6_PP1_r2.fastq.gz, total number of reads: 45002790 uniquely mapped reads: 39416406 read length: 75bp, type: single-end
sample: ChIP-Seq_RXR_PP1_r1.fastq.gz, total number of reads: 102634572 uniquely mapped reads: 86404217 read length: 50bp, type: single-end
sample: ChIP-Seq_LSD1_GT_r1.fastq.gz, total number of reads: 37584097 uniquely mapped reads: 30580387 read length: 75bp, type: single-end
sample: ChIP-Seq_LSD1_GT_r2.fastq.gz, total number of reads: 42597962 uniquely mapped reads: 33055982 read length: 75bp, type: single-end

Antibodies

rabbit anti-H3K27ac Active Motif Cat# 39133, RRID:AB_2561016
rabbit anti-H3K4me1 Abcam Cat# ab8895, RRID:AB_306847
rabbit anti-H3K4me2 Millipore Cat# 07-030, RRID:AB_11213050
rabbit anti-LSD1 Abcam Cat# ab17721, RRID:AB_443964
goat anti-FOXA1 Abcam Cat# ab5089, RRID:AB_304744
goat anti-FOXA2 Santa Cruz Biotechnology Cat# sc-6554, RRID:AB_2262810
goat anti-GATA4 Santa Cruz Biotechnology Cat# sc-1237, RRID:AB_2108747
mouse anti-GATA6 Santa Cruz Biotechnology Cat# sc-9055, RRID:AB_2108768
rabbit anti-HNF6 Santa Cruz Biotechnology Cat# sc-13050, RRID:AB_2251852
rabbit anti-RXRA Santa Cruz Biotechnology Cat# sc-553, RRID:AB_2184874

Peak calling parameters

Bowtie2 v2.2.7 was used to map ChIP-seq reads using the option "--very-sensitive" and all other parameters set to defaults. The findPeaks program from the HOMER suite (v4.8) of bioinformatics tools was used to call peaks with default parameters for transcription factors and the option "-style histone" for histone modifications. Condition- and differentiation stage-matched input samples were used (e.g. ChIP-seq_input_PP2_r1.fastq.gz was used as input for ChIP-seq_LSD1_PP2_r1.fastq.gz). Replicate-matched inputs were used where available.

Data quality

All peaks were called using the default parameters in the findPeaks program within HOMER suite of bioinformatics tools. The default enrichment and FDR for called peaks is 4-fold and 0.001, respectively.

Software

Bowtie2 v2.2.7 was used to map ChIP-seq reads to the hg19 reference genome. The findPeaks, makeTagDirectory, getDifferentialPeaks, makeUCSCfile programs from the HOMER suite (v4.8) of bioinformatics tools were used for peak calling, making tag directories, differential peak analysis, and generating bigWig files for visualizing ChIP-seq data.

Flow Cytometry

Plots

Confirm that:

☒ The axis labels state the marker and fluorochrome used (e.g. CD4-FITC).

☒ The axis scales are clearly visible. Include numbers along axes only for bottom left plot of group (a 'group' is an analysis of identical markers).

☒ All plots are contour plots with outliers or pseudocolor plots.

☒ A numerical value for number of cells or percentage (with statistics) is provided.

Methodology

Sample preparation

Cell aggregates derived from hESCs were allowed to settle in microcentrifuge tubes and washed with PBS. Cell aggregates were incubated with Accutase® at room temperature until a single-cell suspension was obtained. Cells were washed with 1 mL ice-cold flow buffer comprised of 0.2% BSA in PBS and centrifuged at 200 g for 5 min. BD Cytofix/Cytoperm™ Plus Fixation/Permeabilization Solution Kit was used to fix and stain cells for flow cytometry according to the manufacturer's instructions. Briefly, cell pellets were re-suspended in ice-cold BD fixation/permeabilization solution (300 µL per microcentrifuge tube). Cells were incubated for 20 min at 4 °C. Cells were washed twice with 1 mL ice-cold 1X BD Perm/Wash™ Buffer and centrifuged at 10 °C and 200 x g for 5 min. Cells were re-suspended in 50 µL ice-cold 1X BD Perm/Wash™ Buffer containing diluted antibodies, for each staining performed. Cells were incubated at 4 °C in the dark for 1 hr. Cells were washed with 1.25 mL ice-cold 1X BD Wash Buffer and centrifuged at 200 g for 5 min. Cell pellets were re-suspended in 300 µL ice-cold flow buffer.

Instrument

FACSCanto™ (BD Biosciences)

Software

FlowJo v10 software https://www.flowjo.com/solutions/flowjo, RRID: SCR_008520

Cell population abundance

Data for 10,000 events in the post-sorted fraction were recorded for each sample. Purity was determined as falling within gated regions determined to exclude negative control (isotype-stained) samples.

Gating strategy

Negative isotype controls were used for all flow cytometry samples. Each sample was split evenly and stained with isotype controls for the same fluorophore(s) used for the target protein staining. Gating strategy excluded regions detected in isotype controls to reduce background as much as possible for target-protein-stained samples.

☒ Tick this box to confirm that a figure exemplifying the gating strategy is provided in the Supplementary Information.
