# Supplementary material for: LSD1-mediated enhancer silencing attenuates retinoic acid signalling during pancreatic endocrine cell development
Source: Nat Commun. 2020 Apr 29;11:2082. doi: 10.1038/s41467-020-16017-x (PMC7190832; doi:10.1038/s41467-020-16017-x)
Supplement: Supplementary file 25 — Description of Additional Supplementary Files [file 41467_2020_16017_MOESM25_ESM.pdf]

Title: Supplementary Data 1.
Description: Proximal LSD1 ChIP-Seq peaks at PP1 stage.

Title: Supplementary Data 2.
Description: Distal LSD1 ChIP-Seq peaks at PP1 stage.

Title: Supplementary Data 3.
Description: LSD1-bound enhancers deactivated from PP1 to PP2 (G1 enhancers).

Title: Supplementary Data 4.
Description: LSD1-bound enhancers active at PP1 and PP2 (G2 enhancers).

Title: Supplementary Data 5.
Description: LSD1-bound enhancers activated from PP1 to PP2 (G3 enhancers).

Title: Supplementary Data 6.
Description: Distal LSD1 peaks not associated with G1, G2, or G3 enhancers.

Title: Supplementary Data 7.
Description: Genes up-regulated by inhibition of LSD1 during PP1 to PP2 transition (LSD1iearly). All differential expression was determined using DESeq2.

Title: Supplementary Data 8.
Description: Genes down-regulated by inhibition of LSD1 during PP1 to PP2 transition (LSD1iearly). All differential expression was determined using DESeq2.

Title: Supplementary Data 9.
Description: Genes up-regulated by inhibition of LSD1 during PP1 to PP2 transition (LSD1iearly) near G1 enhancers. All differential expression was determined using DESeq2.

Title: Supplementary Data 10.
Genes up-regulated by inhibition of LSD1 during PP1 to PP2 transition (LSD1iearly) near G2 enhancers. All differential expression was determined using DESeq2.

Title: Supplementary Data 11.
Description: Genes up-regulated by inhibition of LSD1 during PP1 to PP2 transition (LSD1iearly) near G3 enhancers. All differential expression was determined using DESeq2.

Title: Supplementary Data 12.
Gene ontology for up-regulated genes near G1 enhancers. Gene ontology enrichments were performed using Metascape.

Title: Supplementary Data 13.
Description: Gene ontology for up-regulated genes near G2 enhancers. Gene ontology enrichments were performed using Metascape.

Title: Supplementary Data 14.
Description: Gene ontology for up-regulated genes near G3 enhancers. Gene ontology enrichments were performed using Metascape.

Title: Supplementary Data 15.
Description: Enriched binding motifs at G1 enhancers compared to G2 and G3 enhancers. Enriched binding motifs were determined using Fisher's exact test, 2 sided, adjusted for multiple comparisons.

Title: Supplementary Data 16.
Description: Enriched binding motifs at G2 and G3 enhancers compared to genome other than G1, G2, and G3 enhancers. Enriched binding motifs were determined using Fisher's exact test, 2 sided, adjusted for multiple comparisons.

Title: Supplementary Data 17.
Description: Distal RXR ChIP-Seq peaks at PP1 stage.

Title: Supplementary Data 18.
Description: Genes down-regulated by extended treatment with retinoic acid (RA) during PP1 to PP2 transition (RAextended). All differential expression was determined using DESeq2.

Title: Supplementary Data 19.
Description: Genes up-regulated by extended treatment with retinoic acid (RA) during PP1 to PP2 transition (RAextended). All differential expression was determined using DESeq2.

Title: Supplementary Data 20.
Description: Gene regulation after late addition of retinoic acid (RA) during PP2 to EN transition (RAlate). All differential expression was determined using DESeq2.

Title: Supplementary Data 21.
Description: Gene expression changes caused by RAlate following prior LSD1 inhibition during PP2 to EN transition (RAlate + LSD1iearly). All differential expression was determined using DESeq2.
